# Latest advance anti-inflammatory hydrogel wound dressings and traditional *Lignosus rhinoceros* used for wound healing agents

**DOI:** 10.3389/fbioe.2024.1488748

**Published:** 2024-11-28

**Authors:** Safia Arbab, Hanif Ullah, Nehaz Muhammad, Weiwei Wang, Jiyu Zhang

**Affiliations:** ^1^ Key Laboratory of Veterinary Pharmaceutical Development, Ministry of Agriculture, Lanzhou, China; ^2^ Key Laboratory of New Animal Drug Project of Gansu Province, Lanzhou, China; ^3^ Lanzhou Institute of Husbandry and Pharmaceutical Sciences, Chinese Academy of Agricultural Sciences, Lanzhou, China; ^4^ Medicine and Engineering Interdisciplinary Research Laboratory of Nursing & Materials/Nursing Key Laboratory of Sichuan Province, West China Hospital, Sichuan University/West China School of Nursing, Sichuan University, Chengdu, Sichuan, China; ^5^ Hebei Key Laboratory of Animal Physiology, Biochemistry and Molecular Biology, Hebei Collaborative Innovation Center for Eco‐Environment, College of Life Sciences, Hebei Normal University, Shijiazhuang, Hebei Province, China

**Keywords:** anti-inflammatory hydrogel, wound dressings, Lignosus rhinoceros, wound healing, wound

## Abstract

Wound healing is a physiological process occurring after the onset of a skin lesion aiming to reconstruct the dermal barrier between the external environment and the body. Depending on the nature and duration of the healing process, wounds are classified as acute (e.g., trauma, surgical wounds) and chronic (e.g., diabetic ulcers) wounds. The latter, often affect millions of people globally, take months to heal or not heal non-healing chronic wounds, are typically susceptible to microbial infection, and are a major cause of morbidity. Wounds can be treated with a variety of non-surgical (topical formulations, wound dressings) and surgical (debridement, skin grafts/flaps) methods. Three-dimensional (3D)-(bio) printing and traditional wound dressings are two examples of modern experimental techniques. This review focuses on several types of anti-inflammatory wound dressings, especially focusing on hydrogels and traditional macro-fungi like *L. rhinocerotis* as agents that promote wound healing. In this study, we introduced novel anti-inflammatory hydrogel dressings and offered innovative methods for application and preparation to aid in the healing. Additionally, we summarize the key elements required for wound healing and discuss our analysis of potential future issues. These findings suggest that *L. rhinocerotis* and various anti-inflammatory hydrogels can be considered as conventional and alternative macro-fungi for the treatment of non-communicable diseases. We summarized the development of functional hydrogel dressings and traditional *Lignosus rhinoceros* used for wound healing agents in recent years, as well as the current situation and future trends, in light of their preparation mechanisms and functional effects.

## 1 Introduction

The treatment of wounds and the therapeutic challenges they provide have become major concerns in healthcare with a significant financial impact on worldwide (Grainger, 2015; Rani and Ritter, 2016). The skin, one of the most fundamental organs in the body that shields us from the harsh external environment, is frequently injured by traumas, severe burns, ulcers, and other wounds that compromise its ability to act as a protective barrier and play an important role in sensory perception. Moreover, these injuries have a significant financial impact on society and have an adverse effect on the emotional wellbeing of the patient (Dabrowska et al., 2018; Hu et al., 2019). As a consequence, it is imperative to find efficient therapeutic approaches to encourage wound healing.

Wound healing is a dynamic and complex process of tissue regeneration and growth that includes four stages (Maksimova et al., 2014). The first one is the coagulation and hemostasis that begins from the micro vascular bed injury and includes fibrin clot formation, degranulation and aggregation (Solomennikov et al., 2019). As a consequence, this leads to involvement of fibroblasts, macrophages and endothelial cells in wound repair (Solomennikov et al., 2019). The second, inflammatory stage, is accompanied by infiltration of the woundand its surrounding tissue with inflammatory cells—granulocytes (specifically, fragmented nuclei neutrophils) during the 24–48 h after injury and monocytes 48–72 h after injury. Next, lymphocytes migrate to the wound area recruited by IgG and interleukin 1 IL-1). The third stage, proliferation, continues from 3 days to 2 weeks after injury, followed by fibrin scaffold replacement by a newly formed granulation tissue as a result of extracellular matrix components (collagens types I and III, laminin 1, nidogen) and glycosaminoglycan synthesis by fibroblasts as well as auto- and paracrine actions of fibroblasts. The final stage is a remodeling phase, involving the maturation and reconstruction of nascent tissues, is the final stage of wound healing (Su et al., 2021; Naskar and Kim, 2020). The maturation process mainly includes the degradation of excess collagen fibers by collagenase, rearrangement of collagen and regression of overgrown capillaries, which may last for months to years. Ultimately, the granulation tissue formed in wound healing evolves into normal connective tissue (Naskar and Kim, 2020; Okonkwo and DiPietro, 2017). Numerous endogenous and exogenous adverse factors can disrupt the physiological healing processes, among which the inflammatory phase is the most susceptible to interference. Wound tissue produces various proinflammatory cytokines and chemokine’s at the initial step in the inflammatory phase, which results in the infiltration of neutrophils and macrophages at injured sites. Neutrophils are required to remove debris and digest invading bacteria through phagocytosis, releasing caustic proteolytic enzymes and producing free radicals in the process of their cleansing activities. Additional cells present in wound sites include macrophages, which mediate angiogenesis, fibroplasia and extracellular matrix (ECM) production, thereby bridging the inflammatory and proliferative phases importantly; moderate inflammation facilitates the removal of necrotic tissue, kills local bacteria and promotes wound healing (Kasuya and Tokura, 2014).

The use of traditional medicine or natural compounds has been of great interest in the pharmaceutical field for many years including the development of wound healing agents (Chin et al., 2018; Lai et al., 2016). For example, herbal extracts have been investigated for their healing properties and developed as advanced wound dressings (Monirul Islam et al., 2022). *Lignosus rhinocerotis* is a medicinal mushroom and traditionally used for wound treatment (Lai et al., 2014), by indigenous communities. Its sclerotium holds various medicinal values including anti-microbial and antiviral activities (Eik et al., 2012), making it a good candidate as a natural wound healing agent. Traditionally, people cut the sclerotium of the mushroom into pieces and boil in water prior to its application for medicinal purposes (Chang and Lee, 2004). Consequently, hot water extraction is used to mimic the conventional *L. rhinocerotis* preparation used for wound healing. Many studies have been carried out to improve the hemostatic effect and quicken the rate at which wounds heal. However, in order to meet additional requirements like infection prevention and easy administration to the affected areas, a new type of wound dressing is still required to improve results (Agarwal et al., 2022).

Hydrogels are generally a series of relatively hydrophilic polymers with the ability to form a three-dimensional cross linked network and preserve a large amount of water. Due to their high water content, hydrogels show similar properties with human body tissue and higher biocompatibility, which makes them suitable for biomedical applications (Zhang et al., 2020). Hydrogels have unique advantages in wound dressings due to their excellent biological, physicochemical, and mechanical properties (Alven and Aderibigbe, 2020), including excellent biocompatibility (Graca et al., 2020), a biological barrier effect blocking harmful factors, such as bacteria, from entering the wound (Norahan et al., 2023), and the ability to deliver cargos that promote wound healing in response to environmental changes (Dimatteo et al., 2018). It is a great concern that antibiotic overuse has led to the development of drug-resistant bacterial strains. Therefore, a variety of antimicrobial hydrogels have recently been synthesized to effectively avoid development of drug resistant bacteria (Farazin et al., 2023). Thus, hydrogels have widespread applications in the field of wound healing, and various functional hydrogels have been developed with adhesive, antioxidant, antibacterial, hemostatic, and tissue regenerative properties (Liang et al., 2021). The great potential of super molecular hydrogels has attracted tremendous interest from researchers and they have been used for numerous biomedical applications in the past decade.

Thermo gels, or thermo-responsive hydrogels, are a subclass of the super molecular hydrogels that gelate via hydrophobic interactions. Thermo gels can undergo a sol–gel phase transition because they are constituted of amphiphilic polymers with both hydrophilic parts and hydrophobic parts (Kim and Matsunaga, 2017). Thermo gels typically refer to thermo-sensitive hydrogels which can form a gel at a higher temperature and return back to a liquid at a lower temperature within a certain temperature range, which is contrary to the conventional melt transition behavior (Dou et al., 2014). This gelation procedure does not need any other assistance or other triggers such as enzymes; thus, it is considered as a benign phase conversion procedure. The lack of toxic crosslinking agents renders it more likely that thermo gels would show intrinsic biocompatibility as an injectable *in situ* hydrogel (Liow et al., 2016). Considering thermo gel polymers used in biomedical application, two main categories can be identified on the basis of biodegradability: Firstly, non-biodegradable thermo gels, which include (1) polyacrylates and (2) Pluronic. Secondly, biodegradable thermo gels, which include (1) polyesters, (2) polypeptides, and (3) polysaccharides (Lanzalaco and Armelin, 2017). To undergo phase transition from room temperature to body temperature, thermo gels that can undergo gelation within the range of 25°C–37 °C are especially valuable in the biomedical field. Due to phase transition at physiological temperatures, thermo gels have been used in diverse biomedical applications (Patel et al., 2018).

The aim of the present work is to comprehensively review the various types of 3D- (bio) printed hydrogel constructs (e.g., bioactive/antibacterial hydrogels, composite hydrogels, cell-laden hydrogels) that have been used in wound healing applications. The constructs are methodically presented in a tabulated form giving detailed information concerning the selected materials (e.g., sodium alginate, gelatin, peptides, etc.), the crosslinking (e.g., ionic, chemical) and (bio) printing methods (e.g., extrusion, digital light processing, etc.), the type of encapsulated bioactive (e.g., growth factors, antibacterial agents).

This review highlights the parameters for wound healing enhancement Anti-inflammatory hydrogel wound dressings and details the scientific findings of macrofungi traditionally utilized for wound rehabilitation. It subsequently focuses on *Lignosus rhinoceros* and reporting on its phytochemicals and pharmacological properties which may be beneficial for wound rehabilitation. The prospects on the potential of *L. rhinocerus* as a wound healing agent are also deliberated as the conclusion to this review.

## 2 Structure of hydrogel

A hydrogel’s network structure of cross-linked polymer chains is its solid state (Ullah et al., 2015). The hydrogel’s molecular weight is infinite due to its 3- D dimensional network structure. The most important molecular properties that determine the hydrogel structure are the mesh size and the molecular weight of the polymer chain between the crosslinks (Ganji et al., 2010). Crosslinking of hydrogels can be obtained by chemical (covalent) and physical (hydrogen bonding/entanglement) crosslinks. The swelling of a hydrogel is mainly defined by the diffusion of water into the hydrogel (Holback and Yeo, 2011). There are three stages to the hydrogel swelling: 1. Primary bound water: the point at which water molecules connect to the hydrophilic group; 2. Secondary exceed water: the water molecules’ interaction with the hydrophobic groups already in place; and 3. Free water: When the body swells to equilibrium, water fills the empty spaces (Gibas and Janik, 2010). The rate of swelling depends on the concentration of polymer and the crosslinking density (Okay, 2010). The high degree of crosslinking density causes a decrease in swelling ratio and it increases the brittleness of hydrogel.

### 2.1 Classification of hydrogels

Hydrogels are classified by source, preparation, ionic charge, responsiveness, crosslinking, and physical properties because of their customizable and repeatable mechanical or physicochemical features. For biomedical applications, a visual representation of this additional classification is given. Its main disadvantage is the lack of biocompatibility with some of the polymers (Ullah et al., 2015).

Poly (ethylene glycol), poly (acrylic acid), poly (ethylene oxide), poly (vinyl alcohol), etc., are some of the synthetic polymers used as hydrogels (Lee and Mooney, 2001). Physically cross-linked injectable PNIPA Am-polyethylene glycol hydrogel improves cellular response, axonal growth and helps in recovering sensory motor function. These fabricated hydrogels could be a suitable candidate for the regeneration of axons (Bonnet et al., 2020). Polyethylene glycol is a biodegradable, biocompatible, and suitable chemical for the healing of spinal cord injuries because it inhibits the degeneration of nerve fibers (Kong et al., 2017). Additionally, a hydrogel composed of polyvinyl alcohol and poly acrylic acid may be an effective biomaterial for vascular access with decreased platelet adherence (Mannarino et al., 2020). Gelatin methacrylate and cellulose derivatives are examples of semi-synthetic polymers (Utech and Boccaccini, 2016). Most hydrogels that combine the benefits of natural and synthetic polymers are semi-synthetic or hybrid hydrogels. The hydrogel exhibits both mechanical and biological activity when it combines natural protein with synthetic polymer material. According to Berkovitch and Seliktar (2017), the hydrogel utilized to encapsulate the anterior root ganglion cells was made with polyethylene glycol and proteins.

## 3 Hydrogels with an advanced wound healing process

Hydrogels have been the material of choice for biomedical applications because of their advantageous physical properties, flexible production methods, mutable structures, and desired biocompatibility. Hydrogels are materials that are permeable and can absorb water up to hundreds of times their dry weight due to their three-dimensional (3D) cross-linked networks. Hydrogels swell in fluids due to thermodynamic force, yet they do not break down (Campbell et al., 2018). The molecular chains that comprise hydrogels are bound together by cross linkers, which can be chemical or physical. There are various categories for hydrogels. For example, the different binding methods of hydrogels allow them to be classified into physical and chemical categories. Physical hydrogels are produced by a combination of physical and ionic interactions. (Dodero et al., 2019), hydrogen bonds (Zhang et al., 2016), entanglement of chains (Yang et al., 2016). In general, physical hydrogels are characterized as reversible when being heated or treated with other stimulations (Stern and Cui, 2019).

In contrast, chemical hydrogels are usually stable due to covalently cross linked networks (Sun et al., 2012). Hydrogels can be classified as natural or synthetic based on where the components came from. Based on their chemical composition, synthetic hydrogels can be broadly divided into five groups: poly (acrylamide), poly (hydroxyl alkyl methacrylates), poly (N-vinyl pyrrolidone), poly (acrylic acid), and poly (vinyl alcohol). Natural hydrogels based on peptides and polysaccharides have been investigated most extensively (Xing et al., 2019). Polysaccharides have abundant and easily obtained from renewable sources, such as (plants and marine organisms) (Kumar et al., 2019). For large-scale production, polysaccharide synthesis is a viable, inexpensive, and simple approach (Gupta et al., 2019). Additionally, polysaccharides’ unique chemical and physical characteristics such as their biocompatibility, biodegradability, and lack of immunological responses—are precisely support their extensive use in biological materials (Yang et al., 2017).

Polysaccharide-based hydrogels have special qualities such a high water retention capacity in addition to the specific qualities of polysaccharides themselves. Polysaccharide-based hydrogels have a flexible structure and network configuration that make them useful for biosensors, wound healing, and diffusion control.

### 3.1 Parameters for wound healing enhancement

The wound healing mechanism is divided into four phases: (i) hemostasis, (ii) inflammation, (iii) proliferation, and (iv) remodeling. A series of complicated intercellular interactions involving a plethora of soluble factors, intercellular matrix components, immune cells, and signaling molecules occurs during these phases (Komi et al., 2020).

### 3.2 Hemostasis

The hemostatic process involves several highly complex biological mechanisms. The small blood vessels and capillaries surrounding the incision first contract an attempt to reduce local blood flow. Subsequently, a variety of physiologically active substances are produced by platelets, including proteases, growth factors, and cytokines, such as TGFβ (transforming growth factor), PDGF (platelet-derived growth factor), IGF, and EGF (epidermal growth factor) (Scopelliti et al., 2022). The development of biofilms at the site of a chronic lesion caused by bacterial invasion may obstruct the proliferation of epidermal and endothelial cells. Life can be in threat in serious cases (Chen et al., 2021).

### 3.3 Inflammation

Wound healing is slowed affected by prolonged inflammation caused by bacterial infection. Therefore, antibiotics are utilized to fight bacterial infections in the wound site and are commonly used as one of the standard treatments for wound care management (Drucker, 2012). Finding an effective treatment is necessary to reduce bacterial colonization and infection, which will subsequently reduce the duration of inflammation and improve the wound healing process (Negut et al., 2018).

#### 3.3.1 Early induction of the inflammatory phase

Neutrophils and macrophages recruitment into the wound area is an essential event in the inflammatory phase for homeostasis maintenance, fighting pathogen invasion, and dead tissue removal. Previous studies have shown that the number of macrophages that migrate and infiltrate into the wound area is at maximum on day 3 post-wounding and remains until day 7. The number of neutrophils that migrated to the wound site was maximum at 12 h post-wounding and continuously declined until day 3. The inflammatory phase progression can be accelerated by early infiltration and elimination of neutrophils and macrophages, which prevent excessive inflammation that may lead to chronic wound (s) and scar formation (Maeda et al., 2011).

### 3.4 Proliferation

The second phase of wound healing is re epithelialization, a process that involves keratinocyte proliferation and migration to ensure an adequate supply of cells to encase the wound. The process of re-epithelialization which eventually leads to wound closure is one of the parameters for consideration of a healed wound. This re-epithelialization process is flawed in chronic wounds. Growth factors, cytokines, integrin’s, keratins, matrix metalloproteinase, chemokine’s, and extracellular matrix (ECM) play key roles in the regulation of keratinocyte proliferation and modulating wound closure (Pastar et al., 2014). Furthermore, studies have shown that several growth factors participate in angiogenesis, including vascular endothelial growth factor (VEGF), angiopoietin, FGF, and TGFβ, and that the production of a temporary matrix in the granulation tissue is facilitated by the involvement of fibroblasts (Darby et al., 2014). Furthermore, fibroblasts differentiate into α-smooth muscle actin-expressing myo-fibroblasts, which initiate the wound closure process (Zhang et al., 2020). Nevertheless, hyper activation of fibroblasts and myo-fibroblasts is associated with abnormal scar formation (Zhang et al., 2020). Moreover, macrophages are also involved in granulation tissue formation, secreting a variety of growth factors (FGF, TGFβ, EGF and PDGF) (Barrientos et al., 2008). There are significant differences in tissue structure and physiological characteristics between oral mucosa and skin that lead to variations in the healing enhancement (Table 1).

**TABLE 1:** The difference in structure, surroundings and healing processes between oral mucosa and skin.

	Oral mucosa	Skin	References
Structures	Epithelial and lamina propria, part of the oral mucosa has sub mucosa	Epidermis, dermis and subcutaneous tissue	Glim et al. (2013)
	Keratinization/Incomplete Keratinization	Hair follicles, sebaceous glands, sweat glands	Qin et al. (2017)
	Salivary glands	Hair follicles, sebaceous glands, sweat glands	Glim et al. (2013)
	Thicker epithelium	Relatively thin epithelium	Glim et al. (2013)
	High proliferation rate of basal cells	Low proliferation rate of basal cells	Gibbs et al. (2000)
	Relatively high degree of vascularization	Relatively low degree of vascularization	
Surroundings	Epidermal moistening	Epidermal dryness	Brand et al. (2014)
	Exposure to saliva	Exposure to air	Brand et al. (2014)
	Subjected to chewing force and tension, continuously exposed to bacteria		Brand et al. (2014)
Healing process	The oral mucosa has less neovascularization than the skin	Fibroblasts are more responsive to stimulus	Dutzan et al. (2017)
	Lower inflammation levels		Szpaderska et al. (2005)
	Saliva contains high levels of healing promoting histone and growth factors		Mak et al. (2009)
	Oral keratinocytes have a proliferative capacity greater than that of skin keratinocytes		Szpaderska et al. (2003)
	Oral wounds exhibit rapid re-epithelialization		Schrementi et al. (2008)

### 3.5 Remodeling

The final stage of healing is remodeling, which is intended to remove unnecessary vessels, fibroblasts, and inflammatory cells (Nourian Dehkordi et al., 2019). Granulation and re-epithelialization are the primary processes involved in wound closure. Granulation tissue is a temporary matrix, nevertheless. Indeed, the shift in the ECM’s composition is a significant aspect of wound remodeling. Consequently, the ECM febrile network’s structure is organized and aligned (Li et al., 2019). Fibroblasts secrete enzymes that degrade the extracellular matrix (ECM), and active protease particularly MMPs control the production of new collagen and the destruction of existing collagen, which plays a role in wound healing (Visse and Nagase, 2003). Subsequently, the prior, transient extracellular matrix (ECM) transforms from a loose fibronectin tissue network to a denser, larger collagen bundle. Type I collagen eventually changes from type III collagen (Finnerty et al., 2016). In addition, collagen alignment tends to remain constant, which strengthens newly formed tissue. Skin appendages that may renew include sweat glands, sebaceous glands, and hair (Feng et al., 2019).

## 4 Application of anti-inflammatory hydrogel wound dressings

Typical wound treatments comprise surgical (e.g., debridement, skin grafts/flaps), and non-surgical (e.g., topical formulations, skin replacement, wound dressings incorporating or not growth factors, bioactive agents, nanoparticles) methods. Modern approaches include growth factors/cytokine therapy, stem cells (SCs) therapy, vacuum-aided wound closure, and three-dimensional (bio) printed wound dressings. Another approach involves the bioengineering of skin substitutes based on combinations of biomaterials, growth factors and cells (Saifullah and Sharma, 2023).

In wound dressing applications, anti-bacterial and anti-inflammatory hydrogels have a good impact. A multifunctional and pH-responsive composite hydrogel made of carboxylates agarose and tannic acid, which is ironically cross-linked with zinc salts for wound healing (Ninan et al., 2016). The pH-responsive property of carboxylate cellulose combined with anti-microbial, anti-inflammatory, anti-oxidant property of tannic acid showed increased compressive strength and anti-bacterial activity. Dextran is another polysaccharide used for synthesizing hydrogel. It helps *in situ* gelation and controlled release of immobilized growth factor with the help of chitosan micro particles and showed better wound healing *in vivo* (Ribeiro et al., 2013). Chitosan is an excellent wound healing material because of its homeostatic nature (Ahmed and Ikram, 2016). A physically cross-linked chitosan hydrogel has the ability to reconstruct the skin of third-degree burn, where they tested in pig dorsal area (Boucard et al., 2007). An injectable hydrogel constructed via disulphide bond cross-linking of thiolated polyethylene glycol and silver nitrate. This hydrogel is loaded with desferrioxamine, an angiogenic drug and this could repair the diabetic wound with its angiogenic activity.

Wound dressings are typically compresses or sterile pads that are applied directly onto the surface of wounds in order to protect them from further injury and promote their healing process.

Required characteristics of wound dressings (Farahani et al., 2021; Yang et al., 2018).

• Absorption of wound exudate;
• Permeability of water vapors;
• Maintenance of a moist wound environment (at the dressing/wound interface), thus
• promoting autolytic debridement and removal of dead tissue;
• No adherence to the wound surface and easy removal without trauma;
• Prevention of microbial (e.g., bacteria, fungi, viruses) transport;
• Enhancement of epidermal migration and promotion of angiogenesis and synthesis of
• connective tissue;
• Exchange of gas between wounded skin tissue and the environment;
• Mechanical strength to protect the body from further injury;
• Biocompatibility/biodegradability and nontoxicity;
• Thermal insulation

### 4.1 Applicable type of wound dressings

Traditional WDs belong to passive dressings and are usually applied to dry and well-cleaned wounds and include gauzes and bandages. Gauze dressings are presented by woven and non-woven cotton fibers, viscose, and polyesters. The main function of these dressings is exudate and fluid absorption from an open wound due to their fibrous structure. For example, Xeroform ™ (non-occlusive dressing) is a petrolatum gauze impregnated with 3% bismuth tribromo phenate and is used to cover dry or exudative mild wounds (Barillo et al., 2017). Bandages are applied as secondary WDs made from natural cotton, wool, cellulose, rayon, polyester, or polyamide. It should be highlighted that cotton bandages could be used for wound cleaning but they shed fibers and stick to the wound surface; they are generally used for dry venous and arterial ulcers. Rayon, polyester and polyamide bandages are non-adherent absorbent secondary dressings that are permeable for liquid and water vapors that do not stick to the wound surface and hence are suitable for granulated wounds with a mild to moderate exudate.

Since traditional WDs cannot provide enough drainage of the wound, they have been replaced by modern dressings, which are characterized by semi-permeability and the presence of a highly absorbent layer. In addition, modern WDs accelerate the formation of granulation tissue and facilitate the migration of epithelial cells from the edges of the wound to its center (Silva et al., 2019). The applicable summaries of wound dressings are presented in Table 2.

**TABLE 2:** Different type of the applicable dressings for mixed etiology chronic wounds.

Wound type	Etiology	Properties	Applicable dressing (Example)	Reference
Diabetic foot ulcer	Neuropathy and lower limb diseases	Insufficient oxygen and blood supply to the wound bed; healing stagnates in the inflammation stage Weak, moderate, or profuse exudation	Semipermeable non-adhesive and adhesive seals presented by foams and hydrocolloids Examples: UrgoStart contact dressing Allevyn, Biatain and Tegaderm dressings	Brumberg et al. (2021)
Pressure ulcers	Local ischemia and tissue injury	Local injury of skin or subcutaneous fat Low–to–moderate exudation	Semipermeable non-adhesive foam dressings and hydrocolloids. Examples: Mepilex Ag, DuoDerm, SignalTM, and DuoDerm ExtraThin	Brumberg et al. (2021)
Burns	Thermal, chemical or Radiation skin injuries	Propensity to secondary infection, wounds with profuse exudation potentially extending to dermallayers, subcutaneous fat, muscles and bone tissue	Occlusive dressings with high absorptive capacities Examples: alginate, chitosane, collagen, hyaluronic acid hydrogels or fibrous dressings able to form a gel under a contact with wound surface (carboxymethyl cellulose Hydrofiber, Algisite M, HemCon Bandage Pro, Hydrofiber)	Brumberg et al. (2021)
Chronic venous ulcers	Lower limb vascular diseases	Blood supply disturbance; pronounced formation of necrotic tissue, abundant exudation on ulcer surface, accompanied by multiple infections	Semipermeable foam Dressings Examples: Mepilex, Allevyn, Contreet Ag, Coloplast	Brumberg et al. (2021)

Modern WDs are usually semi-occlusive or occlusive and presented mainly by synthetic polymers and divided into interactive, advanced interactive, and bioactive categories (Przekora, 2020). Interactive dressings include semi-permeable films and foams, advanced interactive dressings are presented by hydrocolloids and hydrogels, while tissue-engineered skin equivalents belong to bioactive WDs (Panisi et al., 2021; Assis et al., 2021). Dressings such as Granuflex ™, Comfeel ™, and Tegasorb ™ are available as sheets or thin films (Westby et al., 2017). Hydrocolloids are prescribed for full-size and partial wounds with low to medium exudation, wounds with scab formation and that can remain on the wound surface for up to 7 days (Kamiska et al., 2020).

## 5 Different hydrogels used for wound healing dressing

Biocompatibility is a vital requirement for a hydrogel to maintain tissue homeostasis by presenting a suitable matrix without damaging the local tissue during chronic wound healing. Biodegradability and the biodegradation rate of the hydrogels are other important aspects because they provide temporary template during the proliferation of fibroblasts, re-epithelialization and neovascularization, and remodeling of chronic wounds. Bio-adhesively also plays an important role for the long-term stability of the hydrogel dressings around the wound area, improving the homeostatic effect, keeping the wound moist, and absorbing the tissue exudates during healing. Since the prolonged healing of chronic wounds can increase the risk of infection which adversely affect the healing process, antimicrobial hydrogels can be useful to prevent infections. As indicated in Section (Schultz et al., 2011), the inflammatory phase is the main reason for the delayed healing of chronic wounds. Anti-inflammatory hydrogels can shorten the wound healing period by facilitating the transition from the inflammatory to the proliferation stage. Limited oxygen and nutrient delivery to the wound area is another reason for the delayed chronic wound healing. Pro-angiogenic hydrogels can stimulate angiogenesis to deliver the required nutrients and oxygen to the wound bed to accelerate chronic wound healing. The functionality of hydrogels can be further improved by incorporating various types of drugs or therapeutic agents into the hydrogels. Drug or therapeutic agent releasing hydrogels can provide controlled and sustained delivery in response to the environmental stimuli. Some commercial hydrogel dressings in wound healing are summarized applications of these dressings differ depending on their material components.

**FIGURE 1:**
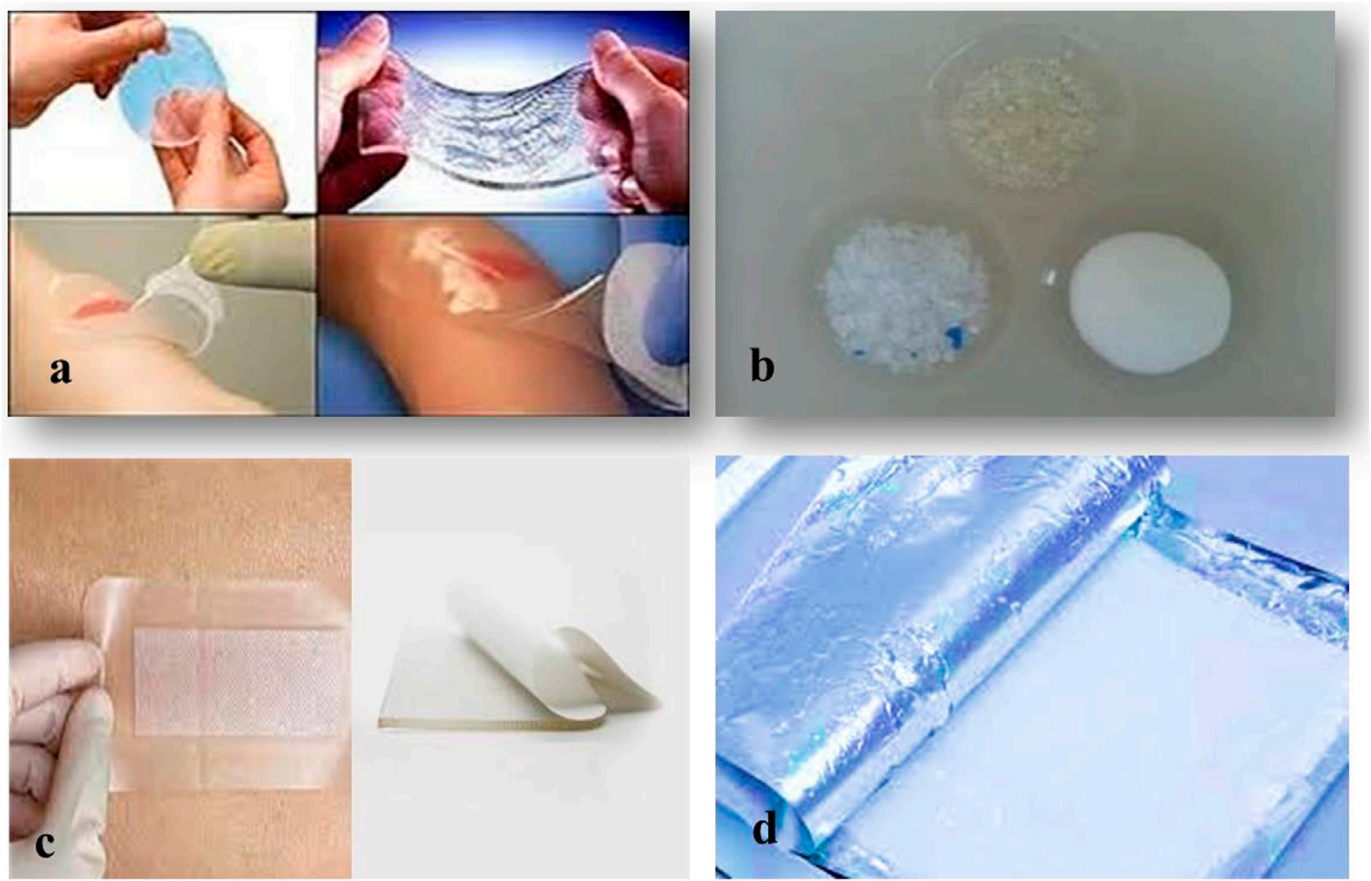
Different forms hydrogel wound dressing available in market. **(A)** Neoheal^®^ hydrogel sheet used for wound dressing, **(B)** Amorphous gel that can be sued for necrotic wounds and burns, **(C)** - Hydrogel film and **(D)** Hydrogel impregnated gauze.

Modern dressings are considered the top choice for curing various wound types due to their exceptional biocompatibility/biodegradability, ability to maintain a moist environment and temperature, pain relief, and improvement of a hypoxic environment. Those most commonly used in clinical practice are films, foams, hydrocolloids, alginates and hydrogels (Nguyen et al., 2023). Figure 1 and Table 3 shows examples of commercially available modern dressings.

**TABLE 3:** Different Commercial hydrogel dressings in wound healing.

Product name	Hydrogel composition Manufacturer	Application	Reference
Aquaderm	2-Acrylamido-2-methyl-1-propanesulfonic acid/2-hydrox 2methylpropiophenone/propylene/glycol/polyethylene glycol Dimethacrylate	Radiation-related chronic wounds/mild burns/pressure ulcers	Firlar et al., 2022; Patil et al., 2019
INTRASITE Gel	CMC/propylene glycol	Chronic wounds	Firlar et al., 2022; Aswathy et al., 2020
MEDIHONEY	Glucose oxidase/Leptospermum Compounds	Mildburns/surgical incisions/various Ulcers	Hixon et al., 2019; Firlar et al., 2022
Neoheal Hydrogel	PEG/PVP/Agar	Mild-burns/various ulcers/chronic Wounds	Firlar et al. (2022)
NU-GEL	SA	Various ulcers	Aswathy et al., 2020; Firlar et al., 2022
Purilon	CA/SCMC	Various ulcers/mild burns	Firlar et al., 2022; Aswathy et al., 2020
Restore Hydrogel	HA	Chronic wounds	Aswathy et al., 2020; Firlar et al., 2022
Simpurity HydroGel	Acrylate/PVA/polyethylene oxide/polyurethane	Mild burns/chronic wounds	Firlar et al., 2022; Aswathy et al., 2020
SOLOSITE Gel	CMC/glycerol	Various-ulcers/mild burns/skin tears	Firlar et al., 2022; Aswathy et al., 2020; Eftekharizadeh et al., 2016
Suprasorb G	Acrylic polymers/polyethylene/Phenoxyethanol	Chronic-wounds/various ulcers/mild Burns	Rippon et al., 2012; Aswathy et al., 2020; Firlar et al., 2022
ActivHeal	It is a primary wound Dressing contains 85% water	Pressure ulcers, leg ulcers, diabetic foot ulcers, cavity wounds	Al-Tabbaa and Ankrah, (2016)
DuoDERM^®^	Hydrocolloid dressing comprising a cross-linked honeycomb matrix of sodium carboxymethyl cellulose, gelatin, pectin and adhesive polymers, and an outer waterproof polyurethane film	Lightly exuding acute (e.g., minor burns, surgical wounds, abrasions and lacerations) and chronic wounds (e.g., stage I–II pressure Ulcers, leg ulcers)	(In et al., 2021)
Nu-Gel™	Transparent, amorphous hydrogel containing sodium alginate	Chronic wound, diabetic foot ulcers, venous leg ulcers And pressure ulcers	Aswathy et al., 2020; Firlar et al., 2022
INTRASITE ♢ GEL (Smith + Nephew)	Clear hydrogel dressing containing modified carboxymethyl cellulose, propylene glycol and water	Shallow, undermined, and deep wounds, granulating cavity wounds, excoriated skin and radiation burns.	Kammona et al. (2024)
HydroTac^®^ (Hartmann) HydroTac^®^ Comfort (Hartmann)	Hydro responsive dressing comprising polyurethane foam combined with AquaClear^®^ Technology HydroTac^®^ Comfort is further coated with a waterproof, bacteria-resistant protective film	Wide range of acute and chronic wounds	Kammona et al. (2024)
PermaFoam^®^ (Hartmann)	Polyurethane foam	Medium to highly exuding, acute and chronic wounds (e.g., diabetic foot ulcers, pressure ulcers II-IV, venous ulcers such as leg ulcers, donor sites, incisions, abrasions)	Atkin et al. (2015)
Mepilex^®^ Ag (Mölnlycke)	Antimicrobial foam dressing with Safe tac technology layer.	Low to medium exuding wounds (e.g., leg/foot ulcers, pressure ulcers). Partial-thickness burns	Atkin et al. (2015)
Tegaderm™ Silicone Foam Dressings	Five-layer polyurethane foam dressing with silicone adhesive	Low to highly exuding wounds (e.g., venous leg ulcers, pressure ulcers, arterial ulcers, neuropathic ulcers, surgical wounds, etc.)	Atkin et al. (2015)
Askina Foam™ (B. Braun, Melsungen Germany)	Polyurethane foam with an outer layer of vapor-permeable and water/bacteria-resistant polyurethane film	Moderate to highly exuding wounds (e.g., pressure ulcers, surgical wounds). First and second-degree burns	Kammona et al. (2024)
ALLEVYN Non-bordered Dressings (Smith + Nephew)	Hydro cellular foam featuring low-tack silicone	Chronic wounds (e.g., diabetic foot ulcers, venous leg ulcers, pressure injuries). Acute wounds (e.g., burns, surgical wounds, donor sites and skin tears)	Kamika et al. (2020)
Tromboguard^®^ (TRICOMED SA, Lodz Poland)	Hydrophilic polyurethane foam combined with an active layer comprising chitosan/alginate, and an outer layer consisting of a semipermeable polyurethane film	Traumatic, postoperative wounds	Kammona et al. (2024)
Granuflex™ (ConvaTec, London, UK)	Hydrocolloid dressing comprising sodium CMC, gelatin, pectin and adhesive polymers	Partial and full-thickness low to moderately exudating wounds Chronic wounds (e.g., stage I-IV pressure ulcers, leg ulcers). Acute wounds (e.g., traumatic wounds such as minor abrasions lacerations), partial-thickness burns and donor sites	Kordestani, (2019)
Confeel^®^ Plus Dressing (Coloplast, Humlebæk Denmark)	Hydrocolloid dressing comprising an elastic/sticky mass encapsulating moisture-absorbing sodium carboxymethyl cellulose (CMC) particles and calcium alginate and covered by a semi-permeable Polyurethane film	Low to moderately exuding wounds (e.g., leg ulcers, pressure ulcers, superficial (partial-thickness) burns, surgical wounds Donor sites and skin abrasions)	Kammona et al. (2024)
Confeel^®^ Plus Transparent (Coloplast)	Hydrocolloid dressing comprising an elastic/sticky mass encapsulating moisture-absorbing sodium carboxymethyl cellulose (CMC) particles and covered by a Semi-permeable polyurethane film	No to low exuding chronic wounds and superficial acute wounds. Superficial (partial-thickness) burns, surgical wounds, donor sites and skin abrasions	Kammona et al. (2024)
Restore Hydrocolloid Dressing (Hollister, New Albany, OH, United States of America	Hydrocolloid dressing	Minimal to moderate exuding partial to full-thickness wounds	Kammona et al. (2024)

Abbreviations: CA, calcium alginate; CMC, carboxymethyl cellulose; HA, hyaluronic acid; PEG, polyethylene glycol; PVA, polyvinyl alcohol; PVP, polyvinyl pyrrolidone; SA, sodium alginate; SCMC, sodium carboxymethyl cellulose.

### 5.1 Film dressings

Film dressings are very thin, transparent polyurethane sheets of increased flexibility, which adhere to the per wound skin and achieve maintenance of moisture in the wound environment. They are impermeable to water and microorganisms but permeable to oxygen, water vapors and carbon dioxide. Due to their strong adherence, they can be applied to moving surfaces like joints but they cause pain and damage to the per-wound upon removal. On the other hand, excessive accumulation of wound fluid beneath the dressing can result in loosening of the adhesive and leakage of the fluid, leading to maceration and facilitating bacterial invasion. Film dressings are typically applied to superficial and newly healed wounds, including graft sites of split skin and peripheral venous catheter sites (Nguyen et al., 2023).

### 5.2 Foam dressings

Foam dressings most commonly consist of polyurethane foam covered by a silicone film which acts as a microbial and water barrier. They are highly absorbing, depending on the foam thickness, texture and pore size, and can maintain a moist wound environment. They also provide thermal insulation. Their increased absorption capacity qualifies them for the treatment of exuding wounds. They can be attached to the wound for up to 7 days, depending on the volume of the wound exudate. They are usually applied to minimal and moderately exuding wounds, burns, chronic wounds and ulcers. Then again, they are not recommended for the treatment of dry epithelial wounds, necrotic wounds and those needing frequent changes [6].

Unlike traditional dressings, such as bandages and gauzes, hydrogel dressings are widely acknowledged for their excellent properties, including mechanical properties that are compatible with biological tissues and exceptional water retention capacity which can keep the wound moist and continuously absorb exudate. In addition, their opportune biodegradation avoids secondary damage during dressing replacement, making them ideal wound dressing materials [74]. Furthermore, compared to other emerging dressings, such as foam and films, hydrogels possess a three-dimensional porous network structure similar to that of a natural ECM, providing a framework for cells to proliferate and migrate. More importantly, hydrogel dressings can be structurally and biochemically designed and functionally integrated to acquire various advantageous properties (Giliomee et al., 2022). Anti-inflammatory hydrogel dressings are foremost representatives. Wound dressing classifications used in this manuscript are presented below in Figure 1 and Table 3; for each type of dressing suitable wounds are briefly named.

### 5.3 Hydrocolloid dressings

Hydrocolloid dressings usually comprise self-adhesive hydrophilic colloid granules (e.g., carboxymethyl cellulose, gelatin, pectin) of various sizes, coated with a water-resistant polyurethane (PU) film protecting the wound from external agents such as bacteria, environmental agents, etc. They are capable of absorbing large amounts of wound exudate while being impermeable to vapors and oxygen, maintaining this way a moist wound environment, stimulating epithelialization and synthesis of collagen, and decreasing the pH of wound fluid resulting in bacteria reduction. In addition, they prevent infection and promote the removal of damaged/infected tissue via autolysis. Moreover, they do not need secondary dressings. They are frequently impregnated with active agents in order to treat lower-extremity and/or pressure ulcers. They are typically applied to low and or moderate exuding wounds, granular and necrotic wounds, as well as acute wounds such as partial and/or full-thickness burns and (post) surgical wounds (Nguyen et al., 2023).

### 5.4 Alginate dressings

Alginates have been extensively used in wound healing because of their valuable properties, like enhanced absorption capability, biocompatibility, non-toxicity and permeability to gases (e.g., oxygen, etc.) and liquids. They have been manufactured in various wound dressing forms, such as films, porous sheets, Nano fibers, hydrogels (Nguyen et al., 2023). In comparison with traditional dressings such as gauze, alginate dressings absorb excess wound exudate while retaining their structural integrity, thus providing a moist wound environment, diminishing bacterial infection and stimulating wound healing (Nguyen et al., 2023). Furthermore, they can diminish wound odor and inflammation, and exhibit hemostatic properties (Nguyen et al., 2023). Upon application onto the wound, alginate forms a gel and easily sloughs when removing the dressing or rinsing with sterile saline. A secondary dressing is usually required in order to stabilize the non-adhesive alginate dressing. Alginate is appropriate for the treatment of both acute and chronic exuding wounds like pressure ulcers, diabetic foot ulcers, (infected) surgical wounds and burns. In the absence of adequate liquid necessary to form a gel, alginate could leave fiber’s at the wound site which could cause inflammation (Nguyen et al., 2023). Alginate dressings are usually combined with various synthetic polymers to increase their mechanical properties. The therapeutic efficiency of the composite dressing is dependent on the ratio of synthetic polymers to alginate, the degree of crosslinking as well as the encapsulation of antimicrobial agents and/or nanoparticles (Zhou et al., 2020). Table 3 shows commercially available modern dressings.

### 5.5 Hydrogel dressings

The development of hydrogels as potential wound dressings for pressure ulcers, dry chronic wounds, necrotic wounds, burns, etc., has received a lot of attention because of their three-dimensional (3D) porous structure mimicking extracellular matrix (ECM), their high water absorption and their mechanical properties (e.g., elasticity, softness) providing a cooling/soothing effect and facilitating the dressing application and removal, their oxygen permeability and their ability to encapsulate various active ingredients (e.g., pharmaceutics, growth factors (Chun et al., 2018; Yuan et al., 2023). In particular, injectable hydrogels have triggered research interest due to their ability to fill irregular wounds, thus avoiding gel fragmentation, and their inherent self-healing properties (Wang et al., 2022).

Both natural (e.g., collagen, chitosan, hyaluronic acid, alginate, gelatin, etc.) and synthetic (e.g., poly (ethylene glycol methacrylate), poly (ethylene oxide), poly (hydroxyethyl methacrylate), poly (acrylic acid), etc.) polymers have been used for the formation of hydrogels (Yuan et al., 2023), with a preference towards natural polymers exhibiting biocompatibility, nontoxicity, enhanced cell attachment and strong activity against bacteria (Wang et al., 2022). Nevertheless, the performance of hydrogels formed using a single natural or synthetic polymer is often limited, so the research interest has focused on the design/development of multifunctional hydrogels with superior properties to be applied as wound dressings (Yuan et al., 2023). including hydrogels efficiently encapsulating conductive agents, which further promote wound healing via regulation of cell activities like adhesion, proliferation and migration (Wang et al., 2022), as well as Gels pharmaceutics, bioactive agents and/or nanoparticles (Yang et al., 2018). It should be pointed out that all hydrogel properties (e.g., physicochemical, rheological/mechanical, biological) can be readily affected by their chemical composition (i.e., selection of polymer backbones, functional groups, crosslinking mechanism and secondary crosslinking interactions, as well as integration of Nano composites) resulting in the formation of hydrogels exhibiting critical characteristics like inject ability, stimuli responsiveness, self-healing (Wang et al., 2022).

## 6 Three-dimensional printing

Hydrogel synthesis by 3D printing is the most advanced technique that involves sophisticated instruments and multistep processes. 3D printing is an expensive technique with controlled quality and pore structure. Limitations of this method are filament resolution (Wang et al., 2022). The 3D-printed porous constructs promote oxygen exchange and ease the removal of metabolites, improving this way of cell proliferation (Tsegay et al., 2022). The introduction of 3D printing to wound dressings has revealed promising results as a consequence of the method’s capability for controlled design and fabrication of dressings exhibiting tuned microstructure (Tsegay et al., 2022). Furthermore, it allows the temporally and spatially controlled release of various bioactive agents (e.g., drugs, growth factors, antimicrobial agents, nanoparticles) (Alizadehgiashi et al., 2021).

### 6.1 Three D-printed hydrogels

Hydrogels are a popular class of biomaterial inks owing to their biomimetic properties and their benign processing conditions entitling them as suitable candidates for TE applications. They are usually printed in the form of their precursor materials and their final structure is obtained via crosslinking during or post 3D printing (Giliomee et al., 2022). Shape fidelity and collapsing are typical challenges in 3D hydrogel printing related to the viscoelastic properties of the ink and its solid content, respectively. Ideally, the ink should be able to flow through the nozzle throughout the printing process and preserve its shape post printing (Leppiniemi et al., 2017). Hydrogel precursors need to have a suitable viscosity to preserve their structural integrity until crosslinking. This can be facilitated by the increase in the polymer concentration, the addition of composites and the use of (near) gel-phase inks such as gelatin solutions which can be printed at a temperature close to their sol–gel transition (near gel-phase inks) and partially crosslinked hydrogels like alginate solutions containing low concentrations of calcium chloride (gel-phase inks) (Tsegay et al., 2022), as well as via the use of rheology modifers such as cellulose nanofbrils, which could improve ink printability and achieve shape fdelity post printing (Leppiniemi et al., 2017).

Recent advances in 3D printing technologies (e.g., extrusion-based 3D printing) permit hydrogel customization according to wound size and depth (Guo and Longaker, 2023), and enable the formation of multi-component hydrogels exhibiting various microstructures and networks of interconnected pores which facilitate the transport of oxygen, nutrients, metabolic wastes (Guo and Longaker, 2023), as well as the temporal and spatial control of bioactive’s release thus promoting bacteria reduction, favoring tissue proliferation and decreasing healing time (Guo and Longaker, 2023).

Various types of polymers, both natural (e.g., sodium alginate (SA), chitosan, gelatin, carboxymethyl cellulose (CMC-Na) (Zhang et al., 2021), and synthetic (e.g., 4-arm PEG (Giliomee et al., 2022), 2-hydroxyethyl methacrylate (HEMA), polyethylene glycol dimethacrylate (PEGDA), poly (acrylic acid) (PAA) (Tsegay et al., 2022), as well as combinations there have been used for Gels 2024, 10, 147 19 of 61 the formation of hydrogel inks. From a chemical point of view, the selected materials should be easily modifed with various chemical groups in order to be cross-linked via different mechanisms (e.g., free radical, ionic, etc.) and functionalized with appropriate molecules (e.g., functional polymers, adhesion peptides, peptides cleavable by proteases). They should also undergo hydrolysis and/or enzymatic degradation, potentially exhibiting inherent antibacterial properties, stimuli responsiveness, etc. Finally, they could permit the formation of a reversible 3D network via dynamic chemical bonding to enable self-healing (Yuan et al., 2023).

Sodium alginate (SA), a cost-effective marine-derived polysaccharide, characterized by excellent biocompatibility, enhanced aqueous solubility and minimal toxicity, has been widely utilized in 3D printing of wound dressings since it can rapidly crosslink with divalent captions (e.g., Ca2+, Mn2+, Ba2+, Cu2+) and absorbs excess wound fluid while it maintains a physiological moist wound environment (Xu et al., 2022). On the other hand, shape infidelity, excessive swelling, rapid degradation rate, low mechanical properties, etc., could hinder its use in the 3D printing of wound dressings (Liao et al., 2023). Nevertheless, modifcation and/or enhancement of SA by combination with different organic/inorganic materials (e.g., various polymers such as gellan gum (Xu et al., 2022), collagen, gelatin, cellulose, etc., and/or nanoparticles) can improve its performance (Kim et al., 2023). In this respect, Wang and coworkers designed and fabricated a bilayer membrane (BLM) comprising a layer of alginate hydrogel printed on a poly (lactic-co-glycolic acid) (PLGA) fbrous membrane mimicking the dermis and epidermis, respectively. The PLGA membrane was found toprevent bacterial invasion and maintain the hydrogel moisture content, while the hydrogel layer stimulated cell adhesion and proliferation.

## 7 Description and ethno-mycological aspects of *L. rhinocerotis*

Medicinal mushrooms have been valued and used since ancient times by the Chinese, Korean, Japanese, Egyptians, and European communities. They are valued not only for the culinary purposes but also for their nutritional and medicinal values (Tan et al., 2009). The greatest attribute of mushrooms, besides their taste, is their peculiar healing properties. Recently, ethnomycological knowledge of medicinal mushrooms for their curative properties is being tapped. *Lignosus rhinocerotis*, however, was only collected from the wild. In the wild, the tiger milk mushroom grows solitary and makes the collection process time and energy consuming. Since the 2000s, large-scale cultivation of *L. rhinocerotis* in a controlled environment was made successful in Malaysia, overcoming the cost and supply problem (Lau et al., 2015a). Commercialization of this mushroom was then made possible and this opened the opportunity to investigate the potential pharmacological and nutraceutical properties of this mushroom.

This mushroom consists of the pileus (cap), stipe (stem), and sclerotium (tuber). Its morphology is unusual for a polypore as the fruiting body (cap and stem) raises from the tuber under the ground, rather than from woody substrate. The cap and stem are woody while the sclerotium is a compacted mass of fungal mycelium containing food reserves. The sclerotium is white and gives a milk-like solution; and it even tastes like milk (Tan et al., 2009). As the irregular shaped sclerotium remains underground, the collection of the mushroom is challenging. Thus, due to lack of samples, limited study is done on this national treasure.

**FIGURE 2:**
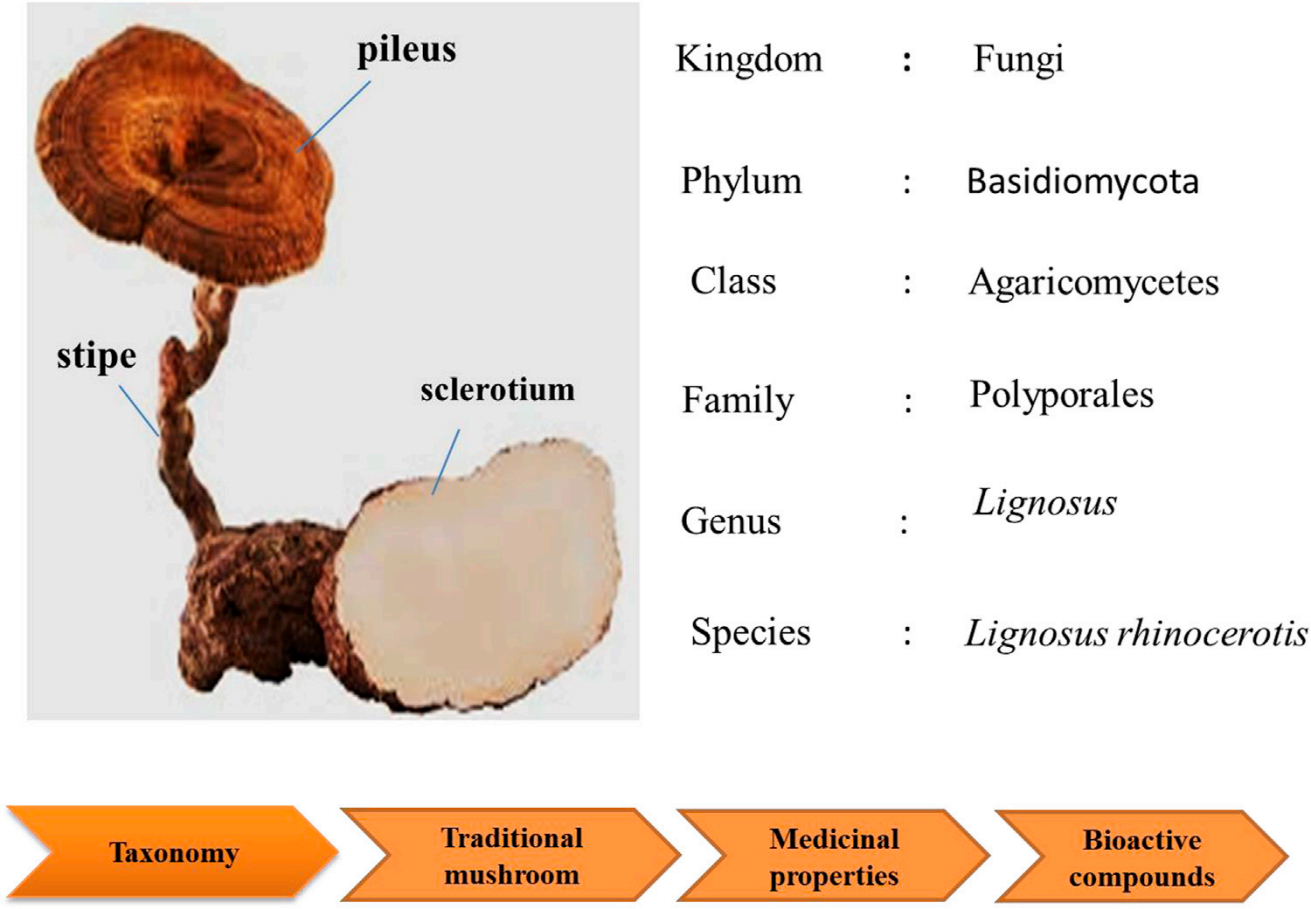
The morphology of *L. rhinocerotis* and its taxonomic classifications.

This mushroom is known as “cendawan susu rimau” which literally means “tiger milk mushroom (Lau et al., 2015b). Early documentation of this mushroom were given by Corner (1989). In the early 20th century, the taxonomy of *L. rhinocerotis* depended primarily on morphological observations. However, in recent years, specimens of “susu rimau” collected in these regions were confirmed as *L. rhinocerotis* on the basis of both micro- and macro morphological characteristics, as well as molecular approaches. To date, L. rhinocerotis is noted to be the “most commonly occurring member *of Lignosus* in Malaysia” (Tan et al., 2015; Lai et al., 2011). Figure 2 shows the taxonomic classification of *L. rhinocerotis*.

According to folklore, it is believed that the mushroom emerges on the spot where the milk of a tigress had accidently dribbled during lactation. The sclerotium of the mushroom resembles the “congealed white mass of milk” (Chang, 2015). Different tribal communities in Malaysia have different referral names, such as “betes kismas” by the Semai (Chang, 2010), “tish am ong” by the Kensiu (Mohammad et al., 2012). *Lignosus rhinocerotis* found in China were called “how gui kou or hurulingzhi” (in Chinese), which means “tiger milk Ganoderma” (Yokota, 2011). In Japan, it is known as “hijiritake” (Lee et al., 2009).

Besides the traditional beliefs that *L. rhinocerotis* was derived from the tiger’s milk, there are many other folklore beliefs about this mushroom. The Semai (indigenous people of Malaysia) believes that *L. rhinocerotis* could reinstate the spirit of a crop and guarantee a lavish harvest. The sclerotia are habitually used during paddy farming and prayer ritual for a bountiful crop yields (Haji Taha, 2006). Alternatively, some crops, for instance, paddy is positioned in a flower-filled container, and suspended over the mushroom (Nallathamby et al., 2018).

The *L. rhinocerotis* are occasionally sold in Traditional Chinese Medicine (TCM). They are used by the TCM practitioner to revitalize the body of the patients. According to Sabaratnam et al. (2013), infusions of *L. rhinocerotis* are said to improve the overall wellness of the individual by enhancing the vitality, energy, and alertness. There are many ways this mushroom is prepared and consumed to treat illness. The mushrooms are grounded or sliced, then boiled with water for drinking or soaked into Chinese wine for external applications (Chang and Lee, 2004). The sclerotium is also eaten raw and with betel leaves to relieve a cough and sore throat. The preparation methods of decoction and/or topical medicine vary among tribes.

## 8 Anti-inflammatory activity of *L. Rhinocerotis*

The anti-inflammatory activity of the sclerotium of *L. rhinocerotis* was previously reported with its hot aqueous, cold aqueous, and methanol extracts (Lee et al., 2012; Lee et al., 2013). Lee et al. (2014), reported that the three extracts of *L. rhinocerotis* exhibited anti-inflammatory properties as shown by the carrageenan-induced paw edema test using Sprague-Dawley rats. The cold aqueous extract, the most potent extract, was subjected to separation by Sephadex G50 gel filtration chromatography. The resulting high-molecular-weight protein fraction was further assessed for anti-inflammatory activity in lipopolysaccharide (LPS), induced RAW 264.7 macrophage cells. The protein fraction was shown to inhibit tumor necrosis factor alpha (TNF-α) production. The anti-inflammatory effect of *L. rhinocerotis* hot aqueous and ethanol extracts on RAW 264.7 cells was further tested (Baskaran, 2015). The ethanol extract showed significant decrease (48.3%–88.5%) of nitric oxide (NO) production from 0.01 to 100 μg/mL dose-dependently but the aqueous extract did not show a significant reduction. The ethanol extract was able to activate signal transducer and activator of transcription 3 (STAT3) pathways by reducing inducible nitric oxide synthase (iNOS) and cyclooxygenase-2 (COX-2) expressions while increasing the interleukin 10 (IL-10) expression. Nallathamby et al. (2016) analyzed the ethyl acetate fraction from the ethanol extract of *L. rhinocerotis*. The fraction significantly reduced the NO production in microglial (BV2) cells by 12%–70% at 10 and 100 μg/mL; respectively. The major compounds of the ethyl acetate fraction were revealed as linoleic acid, oleic acid, and ethyl linoleate. The identified compounds were further tested individually for their anti-inflammatory activities. Treatment with linoleic acid significantly suppressed iNOS and COX2 expression by 1.2-fold as compared to the control. In another study, LPS-induced BV2 cells pretreated with hot aqueous extract (500 μg/mL), n-butanol fraction of hot aqueous extract (250 μg/mL), and ethyl acetate fraction of hot aqueous extract (250 μg/mL), showed maximum inhibition of NO production by 88.95, 86.50, and 85.93%, respectively (Seow et al., 2017). These studies represent the first evidence of anti-inflammatory properties of *L. rhinocerotis* using brain microglial BV2 cells.

### 8.1 Anti-microbial activity of *L. rhinocerotis*

Four extracts of the wild *L. rhinocerotis* sclerotium, i.e., petroleum ether, chloroform, methanol, and aqueous extracts, were screened for their anti-microbial properties (Mohanarji et al., 2012). The four extracts were tested against 15 pathogenic bacteria, including *Staphylococcus*, *Corynebacterium*, *Bacillus*, *Streptococcus*, *Klebsiella*, *Salmonella*, *Pseudomonas*, *Escherichia*, and *Micrococcus* spp.; as well as four fungi species including *Candida* spp. and Mucor sp. Antifungal and antibacterial activities of the extracts were evaluated by measuring the inhibition zone using disc diffusion assay. The methanol and aqueous extracts (30 mg/mL) showed significant inhibition against the tested microbes except for *Streptococcus pyogenes* and *Serratia marcescens*. A qualitative phytochemical analysis showed the presence of alkaloids, protein, gums and mucilage, and flavonoids in the extracts of *L. rhinocerotis* (Mohanarji et al., 2012).

## 9 The utilization of macro fungi for wound healing

**FIGURE 3:**
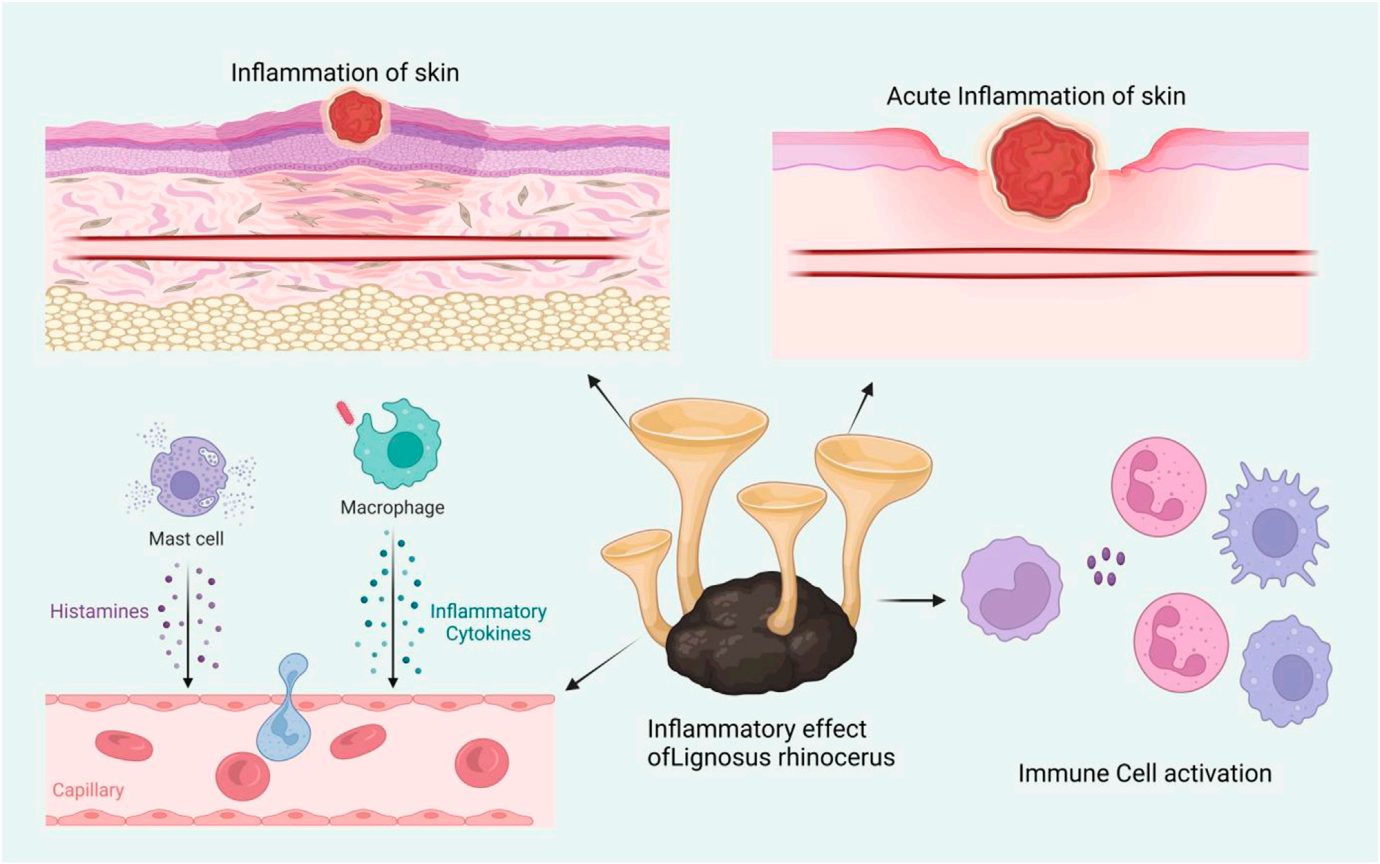
Shows the effect of macro fungi *L. rhinocerotis* studied for their wound healing activity.

Fungi are members of a large, diverse group of heterotrophic organisms which are frequently found living on dead, decaying wood, and other organic matter. They are eukaryotic, with a scope of internal membrane systems, and membrane-bound organelles, and possess a distinct cell wall that is made largely from polysaccharides and chitin (Richardson and Warnock, 2012). The consumption of medicinal mushrooms has long been practiced and there is growing interest in the discovery of bioactive compounds with medicinal values from industries and the scientific communities alike (Pereira et al., 2005). This section provides an overview of the capacities of fungi for wound healing enhancement, both in modern science and traditional knowledge. Figure 3 shows the effect of macro fungi *L. rhinocerotis* for wound healing.

### 9.1 Medicinal mushrooms for wound rehabilitation in the modern age

The use of traditional medicine or natural compounds has been of great interest in the pharmaceutical field for many years including the development of wound healing agents (Chin et al., 2018; Lai et al., 2016). For example, herbal extracts have been investigated for their healing properties and developed as advanced wound dressings (Monirul Islam et al., 2022). *Lignosus rhinocerotis* is a medicinal mushroom and traditionally used for wound treatment (Lai et al., 2014) by indigenous communities in Malaysia. Its sclerotium holds various medicinal values including anti-microbial and antiviral activities (Eik et al., 2012; Yap et al., 2014), making it a good candidate as a natural wound healing agent. Traditionally, people cut the sclerotium of the mushroom into pieces and boil in water prior to its application for medicinal purposes (Chang and Lee, 2004). Therefore, hot water extraction was applied in this study to resemble the traditional preparation of *L. rhinocerotis*.

In addition to that, the wound dressing should provide a hemostatic effect to avoid excessive bleeding. In addition to high antioxidant and anti-inflammatory (Elzayat et al., 2018), the peel extract of traditional medicine or natural compounds exhibits hemostatic activity owing to its high polyphenols content such as ellagitannins (Soltani et al., 2014). Therefore, the combination of both extracts in a wound dressing is anticipated to provide synergistic effects to allow faster healing of acute wounds. Moreover, none utilize the combination both extracts that offers hemostatic and healing properties that may be useful for sport-related wounds that are urgently needed in facilitating the speedy recovery of injured athletes (Scott et al., 2020). Several studies have been carried out for increasing the rate of wound healing and hemostatic effects (Chang et al., 2022; Hu et al., 2022). However, a new class of wound dressing is still required for achieving better outcomes by fulfilling other needs such as the prevention of infections and easy application on the affected areas (Agarwal et al., 2022). Other than offering multiple actions for speedy wound healing, the combination of thermo responsive gels and both extracts is expected to allow self-medication. Table 1 shows the summary of the macro fungi studied for their wound healing activity. The Table 4 showed the Summary of macro-fungi utilized as traditional wound dressing treatment.

**TABLE 4:** Summary of macro-fungi utilized as traditional wound dressing treatment.

Species	Common name	Mushroom part	Application	References
*Lignosus rhinocerus*	Tiger milk mushroom (Wild type)	Fruiting body, tuber (aka sclerotium)	Wound dressing	Mohanarji et al., 2012; Wei et al., 2011
*Handkea utriformis*	Puffball	Fruiting body	Wound dressing	Anusiya et al. (2021)
*Morchella esculenta*	Yellow morel Kann Gitch Batt Gitch	Fruiting body	Wound dressing	Lone et al., 2012; Mahmood et al., 2011
*Fomes fomentarius*	Hoof fungus Tinder Fungus	Fruiting body	Wound dressing	Stamets and Zwickey, (2014)
*Calvatia gigantea*	Giant puffbal	Fruiting body spores	Wound dressing, tea	Anusiya et al. (2021)
*Fomitopsis betulina*	Birch polypore	Fruiting body	Wound dressing	Grienke et al., 2014b; Grndemann et al., 2020
*Lycoperdon pusillum*	Anthua Jatia rutka	Fruiting body	Wound dressing	Anusiya et al., 2021; Rai et al., 2005

In the current studies, extracts of *L. rhinocerotis* were incorporated into topical thermo responsive gel formulations. Phytochemical screening of extracts was first carried out using Gas Chromatography-Mass Spectrophotometry (GC-MS) to identify potential compounds that might contribute to the wound healing effects. Later, all the gels were evaluated for various properties including T gel, texture, rheology, and morphology as well as biological effects (antibacterial and thrombin activities). Wound healing effects of the gels were also evaluated via the *in vitro* cell migration assay for wound (Faris Taufeq et al., 2023).

## 10 Ethno-botanical usage of mushroom preparations for wound rehabilitation

In addition to scientific research on the wound healing activity of fungi, there are a few mushrooms that are traditionally utilized for wound treatment. These macro fungi were reported in the literature for traditional wound treatment practices. Table 5 summarizes the macro-fungi utilized for traditional wound treatment.

**TABLE 5:** Summary Pharmacological relevance utilization of Lignosus rhinocerus to wound healing.

Mushroom part	Origin	Pharmacological Activity	Extraction method	Bioactive/major components	References
Sclerotium	Cultivated	mmunomodulatory activity	Gold water	Polysaccharides	Thuraisingam et al. (2010)
	Cultivated	Anti-inflammatory activity	Hot water/cold water methanol	HMW of CWEprotein components	Ahmed et al. (2011)
	Wild/cultivated	Wound healing activity	Hot water extraction	B-glucan	Lai, (2011)
	Wild	Anti-microbial activity Antibacterial activity	Petroleum ether, chloroform, methanol, and water	Alkaloids, flavonoids, carbohydrates proteins	Lee et al. (2018)

### 10.1 Lignosus rhinocerus

The sclerotium of wild *L. rhinocerus* (Common name: Tiger Milk Mushroom) has been used by Aborigines as a traditional medicine to treat wounds, asthma, fever, breast cancer, stomach cancer, and food poisoning (Tan et al., 2009). The *L. rhinoceros* powder also was mixed with Chinese rice wine and applied topically for treating lumps, sores, and boils (Wong, 2011).

### 10.2 Handkea utriformis

*H. utriformis* (Common name: Puffball) has been used in traditional practices for the treatment of wounds but lacks scientific reports. The fruiting bodies of *H. utriformis* are used in traditional medicine for surgical and burn wound dressings (Petrovi et al., 2019). When the mature fruit body of H. utriformis bursts or is impacted, clouds of brown dust-like spores are emitted and the spore powder is useful to stop bleeding. The practice can be found in the rural state of Europe, North America, and India. A review on “Puffball Usage among North American Indians” has reported that the Indian group from the Missouri River region utilized the puffball as a hemostat. The dried puffball was pulverized and applied to the wound to stop bleeding (Burk, 1983). An extensive survey among the Baiga and Bharia tribes in Madhya Pradesh, central India state, have been showing that the giant puffball was utilized to stop bleeding for healing wounds (Rai et al., 1993). An early report in 1860, reported on the usage of puffball as an anesthetic, like chloroform, for burnt treatment (Wei et al., 2011).

### 10.3 Morchella esculenta

M. esculenta (common name: Yellow morel) has been used in Chinese medicine for thousands of years (Wei et al., 2011). It is also highly utilized by the various tribal group from Kupwara district (Kashmir, India) and Neelum Valley (Azad Jammu and Kashmir, Pakistan). The fruiting body of *M. esculenta* was pulverized to powder for wound application to speed up healing and acts as an antiseptic (Mahmood et al., 2011; Lone et al., 2012).

### 10.4 Fomes fomentarius

*F. fomentarius* (common name: Hoof fungus) was classified by The Greek physician Hippocrates circa 450 BCE as a potent anti-inflammatory agent for cauterizing wounds (Stamets and Zwickey, 2014). In European, West Siberian, and Indian folk medicine, *F. fomentarius* fruit body was made as part of the bandage material. It was pounded with water until it soften and externally applied to wounds to stop the bleeding (Vaidya and Rabba, 1993). In German and Austria, *F. fomentarius* was called a wound sponge or surgical sponge. It is widely used as a styptic by farmers, surgeons, and dentists up to the 19th century (Grienke et al., 2014a).

## 11 Pharmacological relevance of ant-inflammatory hydrogels and *L. rhinoceros* to wound healing

In the last 20 years, a wide variety of biomaterials have been synthesized and used for skin wound healing. However, this is the first time that a native ECM molecule (collagen type I), a biocompatible natural polymer (chitosan), and an inflammation-controlling molecule (dexamethasone) have been combined into a hydrogel that proved to be capable of sustaining mesenchymal stem cells culture, showing that cells remained viable and expressed the anti-inflammatory factor IL-10 upon culture in the hydrogel. Therefore, the next step in our research would be to evaluate the *in vivo* response of hADMSC seeded into the COL1/CHS/Dex hydrogels using a burn animal model.

Due to the declining numbers of wild type *L. rhinocerus*, some institutes or companies were making the initiative on developing techniques for the cultivation of *L. rhinocerus*, such as LiGNOTM Biotech Sdn. Bhd. (cultivar TM02VR), Hong Kong Polytechnic University, and Sanming Mycological Institute (Thuraisingam et al., 2010; Heo et al., 2011) to avoid this valuable mushroom from being extinct. Various in-depth scientific studies were then called for to elucidate the bioactivities of these cultivars.

*L. rhinocerus* was reported to immunomodulation by increasing cytokines (IL-5, IL-6, and MIP-2) expression in RAW 264.7 cells conceivably through the NF-jB/MAPK signaling pathways (Sum et al., 2020). Both signaling pathways have been implicated in corneal epithelial wound healing, scratch injury, and cutaneous wound healing (Thuraisingam et al., 2010; Heo et al., 2011). MIP-2 dominates the early part of the inflammation phase by regulating the migration of granulocytes including neutrophils and stem cells (Reinke and Sorg, 2012). Meanwhile, pro-inflammatory IL-5 and IL-6 cytokines are released to promote inflammation by inducing immune cells to the wound site and concurrently activating growth factors that contribute to angiogenesis and collagen synthesis (Hayta et al., 2018). Increased release of IL-5, IL-6, and MIP-2 caused by *L. rhinocerus* will accelerate the inflammatory phase by early infiltration and elimination of neutrophils and macrophages.

## 12 Conclusion and future perspective

Wound inflammation is complexly regulated. The process of healing can be disrupted by both excessive inflammation infiltration later on and insufficient inflammation levels in the early stages, with the latter occurring more frequently in wound healing. As a result, in recent years, a variety of sophisticated anti-inflammatory biomaterials have been used in wound healing, particularly in the treatment of chronic wounds. One of the most popular of them is the anti-inflammatory hydrogel dressing, which mimics skin functions chemically, physically, and electrically. This review summarizes the scope of emerging anti-inflammatory hydrogel dressings and traditional *Lignosus rhinoceros* used for wound healing agents focusing on wound healing. However, the development of anti-inflammatory hydrogel/*Lignosus rhinoceros* dressings for enhanced wound healing is still in the early stages. Whether previous treatments can be turned into practical applications, these techniques also face specific barriers regarding their biocompatibility and technological outcomes. Unfortunately, the complexity of chronic wound healing in humans cannot be fully replicated in animal models for which therapies have been explored, and human physiology varies significantly from that of mouse Moreover, hydrogel safety needs to be thoroughly evaluated because it can lead to unwanted immunological responses such infections, allergies, and autoimmune diseases. Therefore, extensive study is needed for the development of hydrogel dressings with potential anti-inflammatory properties for effective use in clinical applications.

Future developments in anti-inflammatory hydrogel dressings and the medicinal mushroom *Lignosus rhinoceros* could eventually make it possible to precisely regulate the processes involved in wound healing. Wearable sensors and imaging devices may be used to monitor the level of wound inflammation in real-time and other technologies that include automated stimulus responsiveness may be utilized to adjust therapeutic approaches. Such advances in the development of precise treatment of wounds improve patient curing rates, alleviate pain and reduce costs. Meanwhile, anti-inflammatory hydrogels may also be loaded with other functional components, such as hemostatic, conductive and adhesive materials, making anti-inflammatory hydrogel dressings more powerful and predictable for clinical applications but medicinal mushrooms are low cost as compare to hydrogels, which is a vitally important subject in translational research, particularly in the use of advanced anti-inflammatory hydrogel dressings. Translational research may generate clinically meaningful outcomes in wound healing that improve human health and allow fundamental scientific findings to be translated more efficiently into practical applications. This review has the potential to enhance not just the meaning of wound healing but also the reach of theoretical studies. When developing and innovating new functional hydrogel wound dressings and traditional mushrooms micro-fungi used for wound healing agents, this review can offer new ideas and mechanisms for researchers in the future, advancing the field of wound dressing therapy and expanding the market for functional hydrogel dressings.
